# From protection to predisposition: A narrative review of sex‐specific vascular responses to acute stressors across the lifespan

**DOI:** 10.14814/phy2.71028

**Published:** 2026-08-10

**Authors:** Mya Lax, Jennifer S. Williams

**Affiliations:** ^1^ Michael G. DeGroote School of Medicine McMaster University Hamilton Ontario Canada; ^2^ Cardiovascular Health Research Lab, Department of Kinesiology & Physical Education Wilfrid Laurier University Waterloo Ontario Canada

**Keywords:** acute perturbations, female physiology, menopause, sex differences, vascular function

## Abstract

Cardiovascular disease (CVD) remains a leading cause of morbidity and mortality, with females exhibiting relative biological protection against CVD compared to males through hormonal and nonhormonal mechanisms—a protection that diminishes with age. While previous research has identified early risk factors in CVD pathogenesis, the physiological mechanisms underlying sex and age differences remain poorly understood. To date, no review has synthesized perturbation‐focused studies to clarify sex differences and female physiology, so this narrative review characterized these differences by outlining vascular responses to five acute, commonly encountered perturbations: ischemia–reperfusion injury (IRI), hyperglycemia, hyperlipemia, cold pressor test (CPT), and mental stress. Our synthesis of studies investigating arterial stiffness and endothelial dysfunction, two established early cardiovascular risk factors, reveals sex differences: across perturbations, males respond more uniformly whereas females show greater variability. Specifically, endothelial function responses to IRI and CPT suggest that females may exhibit protection against these perturbations. Most perturbation‐outcome interactions reflect age and hormonal effects, with postmenopausal females often exhibiting the greatest dysfunction. Future research should directly examine sex differences and female‐specific responses after IRI and hyperlipemia. By clarifying sex differences and female‐specific vascular responses to acute perturbations, this work advances understanding of cardiovascular pathophysiology and informs targeted prevention of CVD.

## 1 INTRODUCTION

Cardiovascular disease (CVD), including coronary heart disease, cerebrovascular disease, and peripheral vascular disease, accounts for one‐third of deaths worldwide (Betai et al., 2024; Campbell, 2015). Both modifiable risk factors, such as cholesterol levels, insulin resistance, and psychological stress, and nonmodifiable risk factors, such as sex and age, contribute to CVD onset and progression (Mosca et al., 2011). Importantly, despite sex differences in cardiovascular outcomes being well established, the mechanisms underlying these differences remain poorly understood. Although males and females share most classic CVD risk factors that increase susceptibility to adverse cardiovascular events, their risk profiles differ across the lifespan. Young females appear relatively protected compared to age‐matched males, reflected in lower rates of adverse cardiovascular events, which has been attributed to biological protection in females due to both hormonal and nonhormonal factors (Mosca et al., 2011). Despite this, a rising incidence of hospitalized myocardial infarction (MI) among females aged 35–54, even as rates decline among age‐matched males, has been reported recently (Vaccarino, 2019). Furthermore, females who do experience cardiovascular events face higher mortality and worse prognosis, highlighting sex‐specific variations in vascular physiology that may exacerbate disease severity (Gao et al., 2019).

One prevailing theory attributes sex difference in cardiovascular events to hormonal mechanisms, including alterations in sex hormones estrogen and progesterone. This theory is strongly supported by evidence that the female‐specific advantage against males diminishes with age, likely due to the sharp decline in sex hormones during menopause (Campbell, 2015; El Khoudary et al., 2020; Pardhe et al., 2017; Ryczkowska et al., 2023). Menopause is associated with a worsening coronary heart disease profile, driven by shifts in body fat distribution, increased systolic blood pressure, and hypercholesterolemia, factors that are partially due to declining estrogen levels (Maas & Appelman, 2010). Further supporting this hypothesis, females experiencing premature menopause are at a significantly higher risk of a nonfatal cardiovascular event (Ryczkowska et al., 2023), while oophorectomy has been shown to worsen the overall cardiovascular risk profile (Allison et al., 2008). Overall, the incidence of CVD converges between sexes in older populations, underscoring the importance of hormonal transitions in shaping risk trajectories (Mosca et al., 2011).

Evidence also supports nonhormonal mechanisms behind these sex differences, including intrinsic protection and social factors. Notably, some measures of cardiovascular risk including glycemic indices, inflammatory markers, and vascular reactivity appear independent of hormonal fluctuations, suggesting intrinsic mechanisms of protection in females (Saxena et al., 2012). Hypertension, considered the strongest causal risk factor for CVD by some researchers, differs between the sexes (Fuchs & Whelton, 2020). Although more prevalent in males (Connelly et al., 2022), females develop CVD at lower blood pressure thresholds, increasing their disease burden. In addition, hypertensive females clinically present atypically, which can reduce detection and heighten susceptibility to CVD (Betai et al., 2024; Ryczkowska et al., 2023). Furthermore, female hypertension and CVD are not only under‐diagnosed but also under‐treated. A two‐fold gap in care amplifies female vulnerability to accelerated remodeling and other CVD complications, undermining the cardiovascular protection observed in younger females and highlighting the need for sex‐specific investigation into early, detectable risk factors. Finally, psychosocial stressors, which exacerbate CVD and its risk factors, disproportionately impact females (Dar et al., 2019; Vaccarino et al., 2018). Collectively, these findings illustrate how both biological and psychosocial factors converge to drive sex‐specific differences in CVD outcomes. However, the pathways linking sex, hormonal status, and other risk factors to cardiovascular dysfunction and adverse outcomes remain poorly understood.

Early risk factors such as arterial stiffness and endothelial dysfunction provide insight into cardiovascular pathophysiology. Arterial stiffness is the loss of elasticity and distensibility due to atherosclerotic changes, collagen deposition, and wall thickening (Bonarjee, 2018). Stiffness is a strong predictor of cardiovascular events, potentially because it increases afterload, increasing the work of the heart (Bonarjee, 2018; Kim & Jo, 2024; Vlachopoulos et al., 2010; Willum‐Hansen et al., 2006). Pulse wave velocity (PWV), the gold‐standard measure of arterial stiffness, reflects the speed of pulse propagation along the arterial wall (Segers et al., 2020; Wilkinson et al., 2020). Elevated PWV predicts an increased risk of a first cardiac event (Mitchell et al., 2010). Another measure, the aortic augmentation index (AIx), assesses the reflection of the pressure wave generated by each heartbeat (Einstein et al., 2019). In healthy, elastic arteries, this wave is absorbed and returned, whereas less elastic arteries propagate the wave more rapidly, providing a measure of stiffness. Stiffness increases with age in both sexes, consistently in males throughout the lifespan but only consistently in females after age 50 (Kim et al., 2024). Furthermore, the year following the last menstrual period is a critical period for accelerated increases, highlighting a role for sex hormones in vascular aging (Delgado Spicuzza et al., 2023; Monnier, 1987; Samargandy et al., 2020). Additionally, females have a nearly twofold stronger association between arterial stiffness and mortality compared to males (Regnault et al., 2012), emphasizing the need to investigate female‐specific mechanisms underlying arterial stiffness and cardiovascular risk. Notably, stiffness does not differ in ovarian insufficient females, suggesting that sex differences might not be solely driven by ovarian hormones (Aksoy et al., 2017; Gunning et al., 2020).

Arterial endothelium holds many physiological roles, including regulating blood flow and vessel permeability (Blann, 2007). The loss of endothelial function is therefore associated with pathophysiological cardiovascular risk factors; most notably, impaired endothelial regulation of vessel diameter contributes to the development of hypertension. The gold‐standard measurement of macrovascular endothelial function is flow‐mediated dilation (FMD), determined by the change in artery diameter in response to a transient hyperemia following blood pressure cuff inflation (Fewkes et al., 2022). In functional endothelium, subsequent cuff deflation will trigger a release in nitric oxide (NO), leading to vasodilation (Ras et al., 2013). Notably, FMD impairments are associated with future cardiovascular events (Inaba et al., 2010). Evidence suggests that endothelial function decreases in both sexes with age, but females exhibit better function than age‐matched males until age 70, after which values converge (Skaug et al., 2013). Hormonal contributions are uncertain: one study identified FSH and progesterone, and not estradiol, as mediators of age‐related declines in FMD (Moreau et al., 2020), whereas another implicated estradiol as the driving factor of this change (Wenner et al., 2024). Sex hormones may also acutely influence vascular health, supported by reports of menstrual cycle‐related fluctuations in endothelial function (Brandão et al., 2014; Herrera et al., 2010). Finally, the endothelial dysfunction present in healthy females is more strongly associated with cardiovascular risk factors than in males (Skaug et al., 2014), suggesting that endothelial vulnerability may play a greater role in mediating cardiovascular risk in females.

**FIGURE 1:**
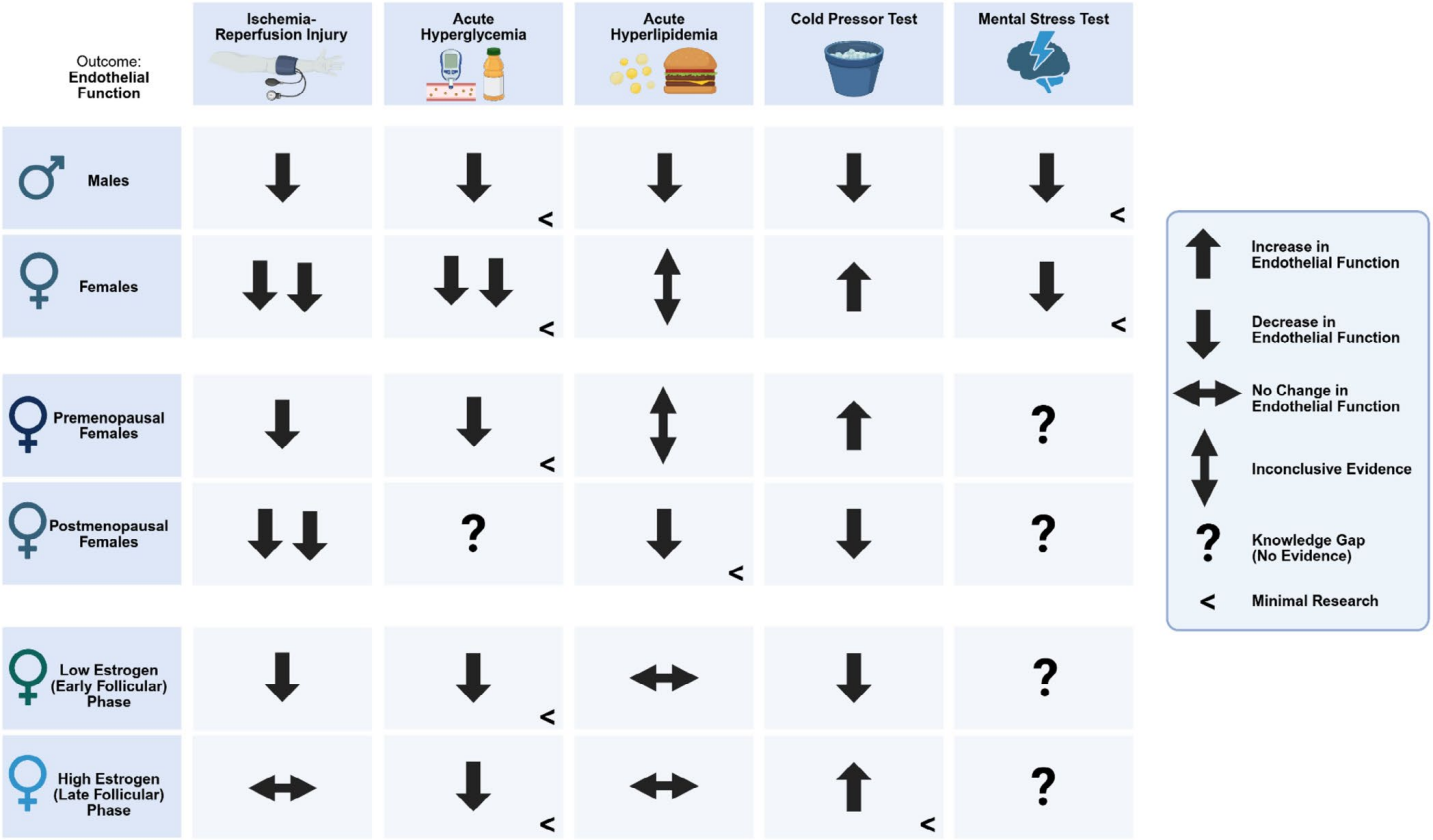
Summary of the effects of each acute perturbation discussed in this review on endothelial function in males and females, pre‐ and postmenopausal females, and females during the low and high estrogen phases of the menstrual cycle. Increases and decreases in endothelial function reflect the change from pre‐ to postperturbation; for example, in males, there is a decrease in endothelial function (worsening function) following the ischemia–reperfusion injury perturbation compared to preperturbation measurements. Double down arrows reflect evidence of greater decrease in endothelial function between comparison groups (e.g., females versus males, post‐ versus premenopausal females). Figure created in Biorender.

**FIGURE 2:**
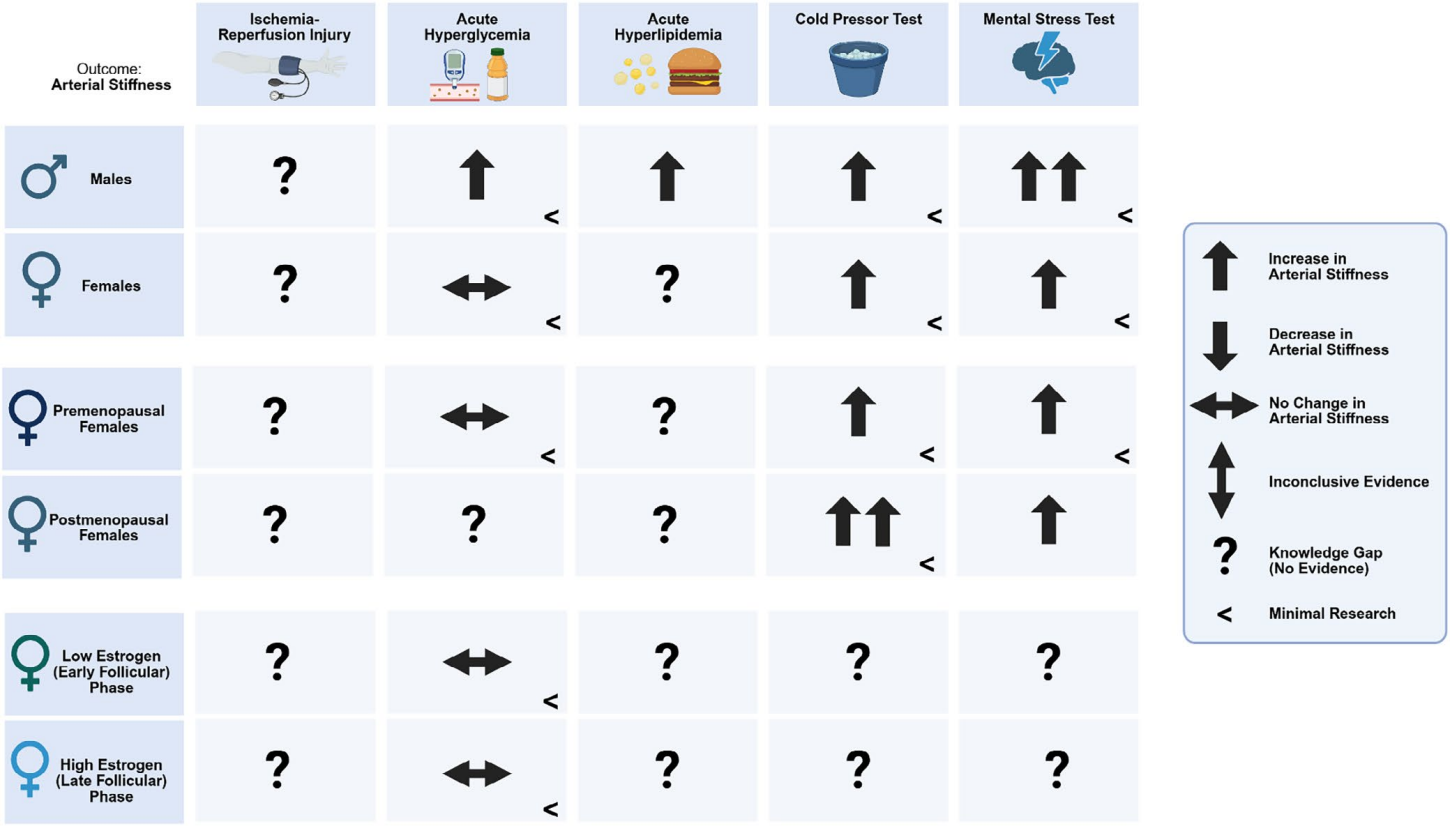
Summary of the effects of each acute perturbation discussed in this review on arterial stiffness in males and females, pre‐ and postmenopausal females, and females during the low and high estrogen phases of the menstrual cycle. Increases and decreases in arterial stiffness reflect the change from pre‐ to postperturbation; for example, in males, there is an increase in arterial stiffness (worsening stiffness) following the acute hyperglycemia perturbation compared to preperturbation measurements. Double up arrows reflect evidence of greater increases in arterial stiffness between comparison groups (e.g., males versus females, post‐ versus premenopausal females). Figure created in Biorender.

**FIGURE 3:**
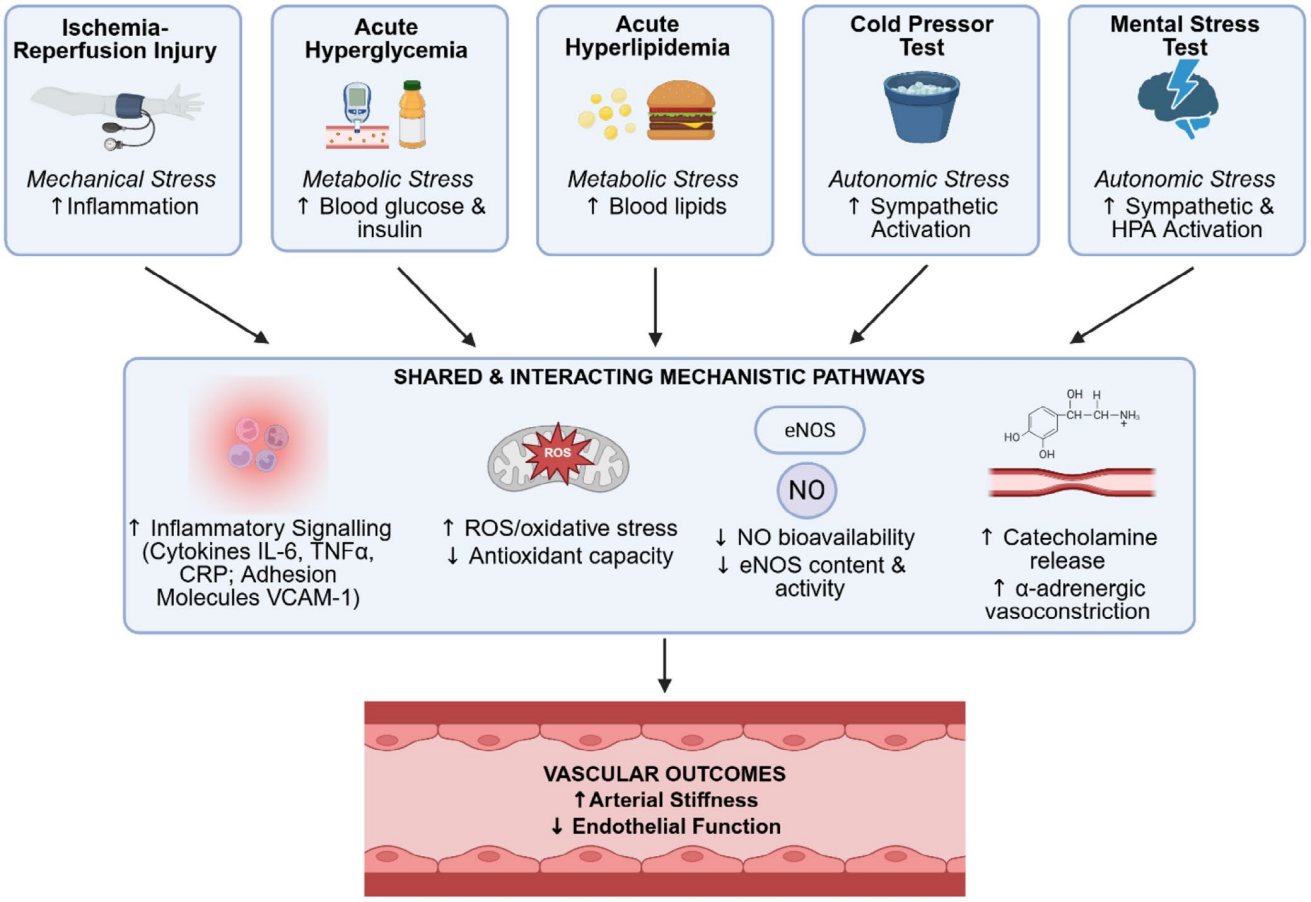
Summary of the potential shared and interacting mechanistic pathways of each acute perturbation discussed in this review, including elevated inflammatory signaling, oxidative stress [e.g., reactive oxygen species (ROS)], decreased nitric oxide (NO) bioavailability via endothelial nitric oxide synthase (eNOS), and autonomic activation resulting in the release of catecholamines inducing vasoconstriction. Figure created in Biorender.

Cardiovascular outcomes can be broadly categorized as macrovascular and microvascular; macrovascular outcomes assess properties of larger conduit arteries and are assessed by direct measures of FMD and PWV, among others, whereas microvascular measures assess the resistance vasculature and are assessed by direct measures of reactive hyperemia index (RHI) and forearm blood flow (FBF) responses to vasodilator, among others. Additional indirect measures of vascular health include surrogate markers of vascular health including blood pressure, pulse pressure, total peripheral resistance, among others. While resting cardiovascular outcomes are well understood and provide valuable diagnostic and prognostic information, gaps remain in our understanding of sex‐specific vascular responses to acute physiological challenges. Perturbations can unmask protective mechanisms or subclinical dysfunction, offering insight into pathways that drive long‐term maladaptive changes. This review therefore compares male and female responses to three groups of controlled stressors, detailed further below: ischemia–reperfusion injury (IRI), metabolic stressors including acute hyperglycemia and acute hyperlipemia, and autonomic stressors including cold pressor test (CPT) and acute mental stress. As this review aimed to characterize human vascular responses to perturbations, animal studies were not included in the initial literature search. However, where evidence from human studies was limited, relevant animal studies were incorporated to provide mechanistic insight into sex differences and female‐specific vascular physiology, and animal studies are clearly identified throughout the review. Although animal research models are essential for studying mechanisms underlying vascular physiology, there are inherent limitations to translating these findings to humans. There are known species differences in cardiovascular anatomy and fluctuations in sex hormones through estrous cycles and menopause timing (Barros et al., 2021; Kehmeier et al., 2022; Koebele & Bimonte‐Nelson, 2016; Lal et al., 2016); thus, animal research included in this review should be interpreted cautiously and considered complementary to human‐derived evidence. This narrative review had two main objectives examining vascular responses to acute perturbations: (1) To characterize sex differences, and (2) To elucidate female‐specific responses across the lifespan, including the influence of sex hormones. This review aims to address gaps in understanding how physiological changes across the lifespan may differently contribute to cardiovascular risk and disease progression. To facilitate comparison across studies, integrated summaries of sex differences, menopausal status, and menstrual phase effects across all perturbations are presented in Figures 1 and 2, along with shared mechanistic pathways in Figure 3, and a summary of included studies is provided in Table S1.

## 2 ISCHEMIA–REPERFUSION INJURY

Ischemia–reperfusion injury (IRI), the paradoxical consequence of reperfusing ischemic tissue, causes extensive tissue destruction (Cowled & Fitridge, 2011) in conditions such as stroke and MI and appears to be influenced by sex (Mandalaneni et al., 2024; Neri et al., 2017). Although females have a higher lifetime risk of ischemic stroke, they appear to be relatively protected against renal IRI, an effect partially attributed to the female hormonal environment (Aufhauser Jr. et al., 2016; Rexrode et al., 2022). Evidence from animal models regarding sex differences in ischemic severity is mixed. Rodent studies generally suggest that sex differences exist in post‐IR infarct size and functional recovery, whereas porcine models report no significant differences between sexes (Kleinbongard et al., 2022). Other animal models report an age‐related increased vulnerability in females mediated by estrogens and support a benefit of 17β‐estradiol in both male and female myocytes against IRI (Booth & Lucchesi, 2008; Hale et al., 1997; Simpkins et al., 1997). By mimicking the oxidative and inflammatory stress occurring in pathological injury by transiently occluding, then restoring blood flow, experimental IRI in animal models demonstrates sex differences in endothelial responses that might underscore clinical differences in ischemic injury in humans.

Few studies have directly examined human arterial stiffness following transient, experimentally induced ischemia–reperfusion (IR), with most studies investigating animal models or using different ischemic perturbations. For example, Pardo et al. demonstrated that female rodent hearts experience less oxidative stress than their male counterparts (Ciocci Pardo et al., 2018). Given that oxidative stress is a predictor of human arterial stiffness, this finding suggests that females might be protected from post‐IR stiffness and injury (Patel et al., 2011). However, this trend is not consistent across species, as mentioned above, and is not supported by human research. Collectively, the lack of human research and conflicting animal studies limit meaningful conclusions, underscoring the need for targeted sex‐stratified human research.

As mentioned previously, the impacts of IR on endothelial function are better represented in the literature and consistently demonstrate differences related to sex, age, and hormonal status. One study reported sex differences in vascular reactivity that varied by arterial location, with FMD in females reduced in some vessels and equivalent to males in others (Nishiyama et al., 2008). Despite this variability, estrogen is consistently shown to exert a protective effect on endothelial function following IRI. Similarly, research by Lalande et al. identified that IR impairs endothelial function similarly in males and females testing in the early follicular phase of the menstrual cycle, having similar levels of circulating estradiol (Lalande et al., 2021). Further, multiple rodent studies suggest that female cardiomyocytes suffer less IRI (Wang et al., 2005), evidenced by greater post‐IR function (Kleinbongard et al., 2022) and reduced infarct size in females (Murphy & Steenbergen, 2007). Supporting these findings, Wang et al. suggested a potential influence of reduced inflammatory response in female rodent hearts on these outcomes (Wang et al., 2005). Based on systemic integration of cardiac and vascular pathways, these sex differences likely extend to the vascular endothelium, where enhanced endothelial function in females might protect against IRI.

This theory is supported by studies showing hormone‐dependent post‐IRI endothelial dysfunction, with estrogen consistently protective during the menstrual cycle. IR precedes blunted endothelium‐dependent vasodilation in the early follicular phase (low estrogen) and preserved FMD in the late follicular phase (high estrogen) (Durchslag et al., 2024; Luca et al., 2016; Van Guilder et al., 2020). This relationship aligns with the clinical increased risk of acute ischemic coronary disease during the early follicular phase, a physiological low estrogen state (Hamelin et al., 2003). Multiple studies propose that activation of estrogen receptors reduces oxidative damage, improving cardioprotection and reducing IRI (Booth et al., 2003; Lin et al., 2009; Wang et al., 2006). This is supported by rodent heart models, where ovariectomy removed female IR protection (Ross & Howlett, 2012) and was associated with an increased risk of ischemic heart disease (Allison et al., 2008).

Further supporting estrogen as a modulator of endothelial function, the post‐IR FMD impairment observed in the early follicular phase is similarly impaired in estrogen‐deficient, postmenopausal females (Durchslag et al., 2024; Parker et al., 2006). Notably, IR produced FMD impairment in premenopausal, early (4 years) postmenopausal, and late (15 years) postmenopausal humans, with the most significant decline in early postmenopause, despite comparable resting values (Delgado Spicuzza et al., 2023). The peak FMD attenuation in this period might be explained by the body's initial inability to compensate for the drop in estrogen levels, to which it gradually adapts during the later postmenopausal period. The observed FMD impairments predict cardiovascular events in postmenopausal females, highlighting the need to explore underlying mechanisms and potential therapeutic targets (Inaba et al., 2010; Rossi et al., 2008). However, it is worth noting that the unanimously reported female protection against IRI conflicts with clinical findings of worse MI outcomes in females, suggesting that response to IRI might not fully account for disease progression (Stehli et al., 2021).

Overall, human and animal models largely exhibit greater protection against IRI in females over males, with reduced infarct size, better functional recovery, and less tissue stiffness, likely mediated by estrogen's effects on oxidative stress, collagen remodeling, and endothelial function. Evidence suggests that this protection varies with age and hormonal status. However, clinical outcomes do not always reflect this advantage, highlighting that IRI response alone may not explain sex differences in cardiovascular disease.

## 3 ACUTE HYPERGLYCEMIA

Acute hyperglycemia, well‐documented to impair the vasculature, is often studied via the strong association of diabetes with cardiovascular disease (Pistrosch et al., 2011). For example, poor glycemic control is more prevalent in females than males with ST‐elevation MI, especially significant in older adults (Otten et al., 2013). However, studies using clamped hyperglycemia and glucose challenge protocols to induce acute hyperglycemia demonstrate potentially maladaptive vascular responses, independent of chronic pathology. This review section highlights that the vascular response to acute hyperglycemia differs based on sex and menopausal status, with postmenopausal females at highest risk.

Clamped hyperglycemia, a technique using a glucose infusion to maintain an elevated blood glucose level (Darden et al., 2020), induces stiffness similarly in both healthy males and males living with diabetes, suggesting this perturbation impacts males regardless of insulin sensitivity status (Gordin et al., 2007). In contrast, a glucose challenge did not significantly impact PWV in premenopausal females, suggesting that acute hyperglycemia does not induce arterial stiffening in this group (Williams et al., 2020), in contrast to males. This relationship persisted in both the early and late follicular phases of the menstrual cycle suggesting that the protective effect in premenopausal females may be independent of short‐term physiologic fluctuations in estrogen levels. In another study, glucose uptake was positively associated with arterial compliance and distensibility in females, but not males (Giltay et al., 1999), which may help explain sex‐specific vascular responses to acute glucose elevations observed in other studies.

To fully understand the sex differences across the lifespan, these findings should be interpreted alongside data from postmenopausal females. This review found no studies directly comparing postmenopausal females to premenopausal females or males in response to an acute hyperglycemic challenge. However, one study in postmenopausal females identified elevations in PWV in response to a glucose challenge in females with normal glucose tolerance, but not with impaired glucose tolerance or diabetes (Lee et al., 2020). Further, one study identified that females demonstrate lower baseline arterial stiffness than males across all glycemic statuses until age 60 (Zhang et al., 2023), when this sex difference reverses in those with normal glucose regulation and is attenuated or lost in those with prediabetes or diabetes. Therefore, relative female protection is influenced by both age and glycemic regulation. The shift at age 60, likely reflecting the menopausal transition, suggests that postmenopausal loss of estrogen may increase susceptibility to vascular changes which could be further exacerbated by acute hyperglycemia.

Studies more frequently measure endothelial function after acute hyperglycemia, but their results are more variable, potentially due to differences in participant characteristics or hormonal status. For example, hyperglycemia decreased FMD in both males and females (Ceriello et al., 2002; Weiss et al., 2008) while paradoxically increasing plasma nitric oxide in females with diabetes, but not in males, supporting the idea that acute hyperglycemia engages sex‐specific endothelial pathways (Har et al., 2014). Further, an oral glucose challenge impaired FMD in premenopausal females, to levels similar to young males (Williams et al., 2019); however, another study reported that females more frequently exhibited impaired FMD as in males with comparable glycemic status (Skaug et al., 2014). The high‐estrogen environment during the late follicular phase of the menstrual cycle did not protect against endothelial dysfunction during the glucose challenge, suggesting other mechanisms beyond short‐term hormonal changes mediate these observed sex differences. Supporting this, despite having greater baseline endothelium‐dependent vasodilation, female rabbit aortas were more susceptible than males' to high‐glucose‐induced endothelial impairment (Goel et al., 2008). Furthermore, while acute estrogen infusion worsened endothelial dysfunction in female rodents, it did not affect male rodents. Given Gilligan et al.'s finding of 17β‐estradiol replacement improving endothelium‐dependent vasodilation in humans, the rodent findings suggest that estrogen's effects can vary depending on the metabolic context (Gilligan et al., 1994). Further, considering the variability between the findings of Williams et al., Skaug et al., and Goel et al., these results highlight sex differences in estrogen's vascular responses to hyperglycemia but may not fully capture the complexities of hormonal changes across the female lifespan. In addition to arterial stiffness and endothelial dysfunction, other vascular outcomes support the observed sex differences described. Clamped hyperglycemia significantly increased systolic blood pressure in females but not males, while circulating angiotensin II remained higher in males, suggesting that blood pressure regulation under acute hyperglycemia differs by sex (Har et al., 2014). Together, these results suggest sex‐specific blood pressure responses to acute hyperglycemia, potentially driving sex differences in cardiovascular outcomes.

Overall, sex differences exist in vascular responses to acute hyperglycemia, with evidence of menopausal status potentially influencing these responses. The divergent responses in arterial stiffness, endothelial function, and blood pressure highlight distinct sex‐specific mechanisms that contribute to cardiovascular risk. While premenopausal females appear protected against hyperglycemia‐induced stiffness and endothelial dysfunction, evidence suggests that postmenopausal sex hormone loss might impact endothelial signaling pathways; however, further research is needed in examining the role of sex hormones and menopausal status in vascular response to hyperglycemia.

## 4 ACUTE HYPERLIPEMIA

Abundant research links long‐term high‐fat intake to cardiovascular disease (Wali et al., 2020), of which females are more susceptible despite generally having a lower risk of cardiovascular events than males (Harris et al., 2012). For example, dyslipidemia disproportionately contributes to hypertension and vascular dysfunction in females (Meagher, 2004), and saturated fat intake has a more harmful impact on females than males (Zong et al., 2016). However, in contrast to these long‐term findings, the vasculature in females appears to be better protected from a single high‐fat meal (HFM), which might better explain the physiological mechanisms modulating paradoxical sex differences in cardiovascular risk (Harris et al., 2012).

The sex‐specific effects of a HFM on stiffness are poorly researched with no identified direct comparisons and few identified investigations in females. This interaction is more frequently investigated in males who show increased postprandial PWV (Fryer et al., 2021). Greater postprandial reductions in AIx were observed in lean and type 2 diabetic males, compared to obese males, introducing dysmetabolism as another important factor that may modulate postprandial responses (Phillips et al., 2010). In contrast to male‐specific data, a mixed population of males and females demonstrated no impact of a HFM on PWV (Pincu et al., 2022), identifying a clear contradiction to the male response discussed. Based on the difference in these two outcomes that might reflect diverging sex‐specific responses, separate analysis of groups or studies specifically comparing male and female responses are needed to confirm this hypothesis.

Studies investigating postprandial endothelial function are more numerous but inconsistent, and some include direct comparisons between sexes that highlight remarkably different results. One mixed‐sex study demonstrated that despite HFM causing significant increases in both macrovascular and microvascular outcomes, postprandial FMD remained unchanged (Raitakari et al., 2000); however, others demonstrated clear FMD impairment after HFM (Phillips et al., 2010; Vogel et al., 1997) (Bae et al., 2001) (Barringer & Sasser, 2011). Upon further investigation, males and females appear to exhibit opposing FMD responses to HFM: a similar HFM perturbation produced FMD impairment in males, indicating endothelial dysfunction (Muniyappa et al., 2011; Vogel et al., 1997), while improvements in FMD in females suggested improved responsiveness. Supporting this sex difference, one study demonstrated no postprandial FMD change in females, but impairment in males, highlighting the potential for female‐specific vascular resilience (Schillaci et al., 2001).

Despite equivalent hormone levels during the menses phase of the menstrual cycle, males still exhibited a postprandial decrease in FMD, while females showed no change (Harris et al., 2012). Moreover, neither progesterone nor estradiol levels are associated with the observed postprandial FMD response, suggesting an inherent, estrogen‐independent mechanism of vascular protection in females. For example, this protection might be explained by differing levels of vasodilatory mediators, with healthy premenopausal females producing higher amounts of nitric oxide (NO) than males (Forte et al., 1998), especially given evidence that NO partially mediates FMD (Harris et al., 2012). Additional hypothesized mechanisms underlying this sex difference include a greater reliance on alternative vasodilatory pathways, such as endothelium‐dependent hyperpolarization (Godo & Shimokawa, 2020), and higher levels of anti‐inflammatory cytokines in females compared to males, both of which may provide estrogen‐independent vascular protection (Bhagat & Vallance, 1997; Chen et al., 2021).

Evidence in postmenopausal females suggests that sex hormones might still influence endothelial responses to HFM. When physiological levels of 17β‐estradiol were restored in healthy postmenopausal females, FMD at rest was potentiated (Gilligan et al., 1994). This finding, coupled with findings by Harris et al. in premenopausal females, suggests that the impacts of estradiol on HFM‐induced vascular changes might depend on exposure duration. In younger adults, short‐term fluctuations in estradiol appear insufficient to explain sex differences in past studies, whereas chronic deficiency in postmenopausal females likely diminishes vascular protection. Further in support of this hypothesis, ablation of endothelial alpha estrogen receptors in rodents reverses the vascular stiffness and impaired endothelial‐dependent dilation induced by a long‐term high‐fat, high‐carbohydrate diet, reinforcing that estrogen signaling has a role in modulating diet‐related vascular outcomes (Manrique et al., 2016).

Evidence also indicates that sex differences in postprandial FMD responses may be influenced by race. While Muniyappa et al. and Swift et al. reported no significant difference in FMD responses of African‐American and Caucasian females (Muniyappa et al., 2011; Swift et al., 2013), Marinos et al. demonstrated more impaired FMD in African‐American females post‐HFM, resembling the response observed in studies investigating males (Marinos et al., 2015). However, both studies have limitations that impact this comparison: the HFM introduced by Muniyappa et al. was equally high in carbohydrates as in fat, introducing a potential insulin‐dependent mechanism of vasodilation that could have confounded results, and Marinos et al. did not compare findings to a control group. Therefore, future work should focus on the potential attenuation of race on sex differences in vascular responses to HFM (Fewkes et al., 2022; Muniyappa et al., 2011).

Together, these findings suggest that females demonstrate a greater resilience to postprandial endothelial dysfunction than males, likely mediated by intrinsic vascular mechanisms rather than circulating hormone levels alone. However, the loss of resilience after menopause and the responsiveness to estradiol replacement suggest that estrogen might enhance vascular function through mechanisms. Finally, intersections of sex with other factors of identity, such as race, may further impact acute responses to HFM and other vascular challenges; further research examining sex‐race intersections is needed.

## 5 COLD PRESSOR TEST

Sudden cold exposure activates the sympathetic‐adrenomedullary axis, triggering norepinephrine release (Silverthorn & Michael, 2013), resulting in arteriolar constriction, increased heart rate, and increased cardiac contractility. Norepinephrine binding adrenergic receptors on peripheral vasculature and subsequent vasoconstriction (Smith & Maani, 2024) illustrates vascular reactivity: the ability of blood vessels to dilate or constrict in response to stimuli (Navedo et al., 2025). The cold pressor test (CPT), which leverages this response, can be used as a predictor of hypertension, where those with hyperreactivity have an increased risk (Zhao et al., 2015). Exaggerated blood pressure reactivity to CPT is further associated with future cardiovascular events (Lloyd‐Jones, 2022; Zhang et al., 2013). These clinical sex differences have prompted investigations into whether vascular responses to CPT also diverge between males and females.

Evidence largely supports males and females having similar increases in arterial stiffness in response to CPT. Firstly, one study demonstrated that both sexes exhibited comparable CPT‐induced increases in arterial stiffness (Gentilin et al., 2025). However, this study noted that young females had lower overall stiffness at baseline and during CPT, reflecting a more favorable vascular profile consistent with sex‐difference findings from other perturbation studies. Similarly, another study reported comparable AIx increases post‐CPT between sexes, with blood pressure contributing only in males, suggesting sex‐specific mechanisms underlie the similar stiffness responses (Prodel et al., 2018). This is further explored in the mental stress section of this review detailed below, where studies also indicate comparable overall responses with sex‐dependent underlying mechanisms (Chen et al., 2012; Gentilin et al., 2023; Logan et al., 2020).

Given that at baseline, young females have lower arterial stiffness than young males, of high interest is the impact of sex hormone levels on this relationship to ultimately understand implications on menstrual cycle and menopausal status (Gentilin et al., 2025). Menopause is associated with higher baseline arterial stiffness (Samargandy et al., 2020), but no studies have examined how menopausal status or menstrual cycle affect CPT‐induced changes. Postexercise muscle ischemia, a perturbation theorized to activate the sympathetic nervous system similarly to CPT, resulted in greater AIx increases in older (>60 years) compared to younger (<60 years) postmenopausal females (Figueroa et al., 2015). This age difference is supported by preserved resting cardiovascular responses observed in the early postmenopausal period and deterioration in the late postmenopausal period, though no acute perturbation was investigated in this study (Moreau et al., 2012). Importantly, these findings suggest that age‐ or menopause‐related vascular remodeling may amplify sympathetic increases in arterial stiffness, even when baseline hormone levels are similarly low.

Sex differences have also been identified in CPT‐induced vascular responses, especially in vasoreactivity. In males, CPT elicits a significant rise in total peripheral resistance through α‐adrenergic vasoconstriction, whereas females paradoxically vasodilate, likely due to heightened β2‐adrenergic activity that counteracts vasoconstriction (Fatima et al., 2020; Gentilin et al., 2022, 2025; Joyner et al., 2016; McLean et al., 1992; Patel et al., 2014; Pickering & Gerin, 1990). Despite males exhibiting greater blood pressure responsivity, no sex differences were observed in post‐CPT release of norepinephrine (Hussain & Maani, 2023; McLean et al., 1992). The authors suggest that future work examining epinephrine levels, which may better explain these sex‐specific vasoactive mechanisms. Supporting this, males are found to activate greater and longer peripheral vascular responses, while females preferentially activate cardiac responses to CPT, including heart rate and myocardial blood flow, although some measurements conflicted between studies (Kilgour & Carvalho, 1994; Vandavasi & Sukumar, 2016). Additionally, in males, AIx correlates with total vascular resistance post‐CPT, linking α‐adrenergic vasoconstriction to both endothelial function and arterial stiffness, whereas these processes appear independently regulated in females (Prodel et al., 2018).

One study demonstrated that cyclical hormonal fluctuations might influence vascular reactivity, with high‐estrogen phases attenuating post‐CPT vasoconstriction (Jacob et al., 2022), suggesting that estrogen may play a multifactorial role in modulating vasoactivity (Fatima et al., 2020). Moreover, since progesterone induces norepinephrine release, mid‐luteal phase testing may unmask sex differences that are obscured during the follicular phase. Across the menstrual cycle, autonomic balance appears to shift towards sympathetic predominance during the luteal phase (Jasrotia et al., 2018), reflected by greater overall cardiovascular reactivity to CPT (Tanaka et al., 2003), other perturbations, and at rest (Adkisson et al., 2010; Oldham et al., 2024). Ultimately, these findings suggest a closer consideration of menstrual phase and menstrual status in female cohorts.

Evidencing sex hormones as regulators of sympathetic activation, females taking combination oral contraceptives responded to CPT with greater vasodilation and prolonged increase in heart rate than in any menstrual phase, although blood pressure increased among groups similarly (Jacob et al., 2022). These findings suggest that hormonal status, whether endogenous or exogenous, modulates the mechanisms driving vascular responses to sympathetic stress. Further, a maximal voluntary end‐expiratory apnea protocol, a stressor similar to CPT (Smith & Maani, 2024), provoked vasodilation in premenopausal females but not in postmenopausal females, suggesting that the loss of ovarian hormones reduced vascular resilience to acute sympathetic activation. Further, pain responses to CPT also vary across the menstrual cycle, with higher progesterone levels linked to greater pain intensity and increasing estradiol attenuating this relationship (Jasrotia et al., 2018; Stening et al., 2007). Together, these findings underscore that sex hormones shape both cardiovascular and pain responses to sympathetic stressors.

Overall, CPT elicits robust sympathetic activation that provokes different responses based on sex and hormonal status, with peripheral adjustments dominating in males and cardiac adjustments dominating in females. While males and females exhibit similar CPT‐induced arterial stiffness increases, females may have peripheral artery protection. Sex differences are also evident in endothelial responses to the CPT, reflected by diverging vasoactivity responses and predominating receptors. Hormonal fluctuations across the menstrual cycle and with menopause influence these responses, underscoring the importance of accounting for sex and hormonal status when assessing sympathetic and vascular function using CPT.

## 6 MENTAL STRESS

Mental stress is a recently emerging cardiovascular risk factor that operates independently of conventional risk factors (Mehta et al., 2022; Vancheri et al., 2022). Mental stress can manifest acutely, such as in response to a natural disaster, or chronically, such as in response to repeated stressors including socioeconomic status. The resultant sympathetic response can induce transient myocardial ischemia (Vancheri et al., 2022). This condition is referred to as mental stress‐induced myocardial ischemia (MSIMI) and can result in angina, MI, arrhythmias, and left ventricular dysfunction. MSIMI occurs more frequently in young females compared to similarly aged males and older females, with some studies suggesting a two‐fold increase in risk (Wokhlu & Pepine, 2016; Jiang et al., 2013; Vaccarino et al., 2018). This suggests that age, sex, and hormonal factors may mediate ischemia induced by mental stress.

Acute mental stress can be experimentally induced using mentally stressful stimuli (MSS) protocols such as the Trier Social Stress Test, mental arithmetic tasks, and public speaking challenges. These stimuli immediately activate the sympathetic‐adrenal‐medullary axis, leading to a rapid release of norepinephrine and epinephrine, and gradually activate the hypothalamic–pituitary–adrenal axis, leading to a slower release of cortisol (Logan et al., 2020). The resulting neuroendocrine changes can alter vascular function, allowing for examination of potential sex‐specific differences in arterial stiffness and functional endothelial responses to acute mental stress.

Studies in males and females both report significant increases in PWV and/or AIx after MSS, although the magnitude may differ by sex and artery type (Kume et al., 2020; Logan et al., 2020). In a mixed‐sex sample, a sustained increase in PWV and AIx was observed during a comparable stimulus, and although sexes were not analyzed independently, this result supports the previously reviewed findings. Studies comparing sexes report conflicting findings: Gentilin et al. report similar central arterial PWV increases across sex groups after MSS, whereas Chen et al. report a greater peripheral PWV increase in male adolescents compared to females (Gentilin et al., 2023; Chen et al., 2012). The conflicting results between studies may be partly explained by differences in artery type: central arteries assessed by Gentilin et al. may respond similarly in males and females, while peripheral arteries assessed by Chen et al. appear more sensitive to sex‐specific differences. Supporting this notion, a female‐only study found that autonomic changes were not associated with stiffness outcomes (Logan et al., 2020), challenging the sympathetic activation mechanism previously described by Vancheri et al. and suggesting either a sex‐specific response or an independent pathway. Altogether, mental stress increases arterial stiffness in both males and females, while also highlighting that the magnitude and mechanism of the response may differ by sex and artery type.

In a mixed sex sample, age had no effect on carotid stiffness during MSS (Lipman et al., 2002). Carotid stiffness was correlated with changes in heart rate during the task and inversely correlated to baroreflex sensitivity. This finding suggests two mechanisms by which stress may alter arterial stiffness: autonomic modulation of heart rate and baroreflex‐mediated vascular adjustments that appear to occur independently of age. Additionally, given the study's older age range (51–86 years) likely represents mostly postmenopausal females, these results might not generalize to younger, premenopausal females who generally have lower baseline arterial stiffness. In this case, observed independence from age may not apply to premenopausal females, and the relationship between stress, heart rate, baroreflex function, and arterial stiffness could differ in these groups. Therefore, future investigations should consider participant menopausal states when selecting for age.

Mixed sex studies confirm MSS impairs endothelial function (Lind et al., 2002; Lima et al., 2019; Sarabi & Lind, 2001) through hormonal and sympathetic mechanisms (Poitras & Pyke, 2013). Among the sex‐specific post‐MSS FMD studies in healthy participants reviewed, only males were investigated. In males, mental stress yielded a reduced FMD that returned to baseline at 4‐h post‐MSS (Ghiadoni et al., 2000), which a male monkey model suggests is driven by endothelium‐derived relaxing factor (Williams et al., 1993). Potentially comparable studies of females were confounded by health status, perturbation type, or other factors, emphasizing the need to prioritize female FMD responses to MSS in future works. Other sex‐specific vascular responses to MSS appear to diverge, with females exhibiting adverse microvascular effects rather than macrovascular and systemic hemodynamic adaptations in males (Martin et al., 2008; Sullivan et al., 2018). Although Harris et al. and Vaccarino et al. reported no sex differences in MSS‐induced FMD impairment (Harris et al., 2000; Vaccarino et al., 2018), Martin et al. observed female‐specific reductions in reactive hyperemia, indicating that acute mental stress preferentially impairs microvascular function in females (Martin et al., 2008). The endothelial dysfunction observed is particularly pronounced in females with blunted vasoreactivity and has been associated with adverse cardiovascular events, highlighting the clinical relevance of microvascular vulnerability (Martin et al., 2008). Supporting this, Vaccarino et al. further outline females' heightened susceptibility to vasoconstriction‐mediated MSIMI, reinforcing the importance of considering sex‐specific mechanisms in acute stress responses (Vaccarino et al., 2018).

Notably, our review identified no studies examining menstrual cycle and MSS. However, reports outline that resting mean arterial pressure, heart rate, and muscle sympathetic nerve activity do not differ across the menstrual cycle, but muscle sympathetic nerve recovery is prolonged in the mid‐luteal phase compared to the early‐follicular phase (Carter & Lawrence, 2007). This suggests that hormonal fluctuations, especially progesterone elevations during the luteal phase, might influence sympathetic activity during MSS. Given the interplay of sympathetic tone and endothelial function, these results indicate that menstrual phase‐specific hormones could modulate microvascular responsiveness after stressors. It is worth noting that populations exposed to chronic stress may respond differently to acute mental stress. Evidence suggests that MSS does not perturb the cardiovascular system in individuals with chronic stress exposure, seen in males with panic disorder and females with depressive disorders, highlighting the importance of considering baseline stress when assessing acute cardiovascular reactivity (D'Urzo et al., 2019; Sullivan et al., 2018).

In conclusion, sex differences exist in vascular responses to MSS, most notably in endothelial function. Females predominantly exhibit microvascular dysfunction whereas males primarily demonstrate hemodynamic adjustments. This divergence contributes to females' heightened risk of vasoconstriction‐mediated MSIMI and underscores the need for sex‐specific research to guide targeted interventions. Furthermore, the attenuated sympathetic response to MSS in females, particularly in their early‐follicular phase, might explain their reliance on microvascular adjustments rather than systemic hemodynamic changes.

## 7 CONCLUSION

This review synthesizes evidence of differences in arterial stiffness and endothelial function in response to acute cardiovascular perturbations to understand sex differences and female‐specific responses across the lifespan. Overall, females demonstrated relative protection in stiffness responses to acute hyperglycemia and endothelial responses to IRI and CPT, with no perturbation‐outcome relationships favoring males (Figures 1 & 2). Endothelial function decreased in males but increased in females after IRI and CPT. These findings align with the previously discussed notion that males may be biologically predisposed to adverse cardiovascular events. However, many conclusions, particularly pertaining to stiffness, were supported by limited evidence, underscoring the need for further investigation.

Menopausal state and menstrual cycle were implicated in many relationships assessed, with evidence nearly unanimously linking low‐estrogen states to worse vascular outcomes after perturbations (Figures 1 and 2). The protective effects of estrogen were most evident in response to IRI, though discussed across multiple perturbations. Notably, some perturbation‐outcome relationships lacked data surrounding menopausal females, including stiffness responses to acute hyperglycemia. Considering diabetes in females is associated with worse CVD outcomes than males, this finding encourages future research to investigate short and long‐term glycemic adaptations (Betai et al., 2024). In addition, only one study assessed the influence of oral contraceptives on vascular responses to perturbations. Given the widespread use of contraceptives, this represents a critical area for future research.

Moreover, reliance on cross‐study comparisons between male‐ and female‐specific cohorts to understand sex differences introduced methodological inconsistencies; therefore, future prospective studies performing a priori sex‐based comparisons are needed. Targeted research that directly assesses sex, hormonal status, and age within each perturbation will be invaluable to understand early, detectable, and modifiable cardiovascular risk factors, thereby informing CVD prevention strategies.

## AUTHOR CONTRIBUTIONS

**Mya Lax:** Conceptualization; data curation; investigation; methodology. **Jennifer S. Williams:** Conceptualization; funding acquisition; project administration; resources; supervision.

## FUNDING INFORMATION

M.L. and J.S.W. are supported by a Natural Sciences and Engineering Research Council of Canada Discovery Grant to J.S.W. (RGPIN‐2025‐05856).

## ETHICS STATEMENT

No ethical approval was required for this narrative review, as it does not present original human or animal research and only relies on existing literature.

## Supporting information


**Table S1.** Characteristics of studies examining mixed‐sex or female‐specific populations only assessing impact of perturbations on measurements of endothelial function and arterial stiffness. Perturbations included ischemia–reperfusion injury (IRI). Outcomes included forearm blood flow (FBF), forearm vascular conductance (FVC), flow‐mediated dilation (FMD), augmentation index (AIx), pulse wave velocity (PWV), systolic blood pressure (SBP), diastolic blood pressure (DBP), heart rate (HR), index of endothelial function (IEF), brachial artery diameter and blood flow (BABF), and total peripheral resistance (TPR).

## Data Availability

No new datasets were generated or created for this review; references to existing original data are provided within this review.
