# Supplementary material for: From protection to predisposition: A narrative review of sex‐specific vascular responses to acute stressors across the lifespan
Source: Physiol Rep. 2026 Aug 10;14(15):e71028. doi: 10.14814/phy2.71028 (PMC13455682; doi:10.14814/phy2.71028)
Supplement: Supplementary file 1 [file PHY2-14-e71028-s001.docx]

| **Author (Year)** | **Mixed-Sex or**  **Female-Only?** | **Population** | **Perturbation** | **Outcomes; type of outcome (macro vs microvascular) where applicable** | **Main Findings** |
| --- | --- | --- | --- | --- | --- |
| **Perturbation: IRI** | | | | | |
| Parker et al. (2006) | Female-Only | Young (aged 20-30, n=14) healthy females  Older (aged 60-79, n=14) healthy females | IRI | FMD; macrovascular  Endothelium-independent dilation; macro | ↓ FMD in older females  ↓ endothelium independent dilation in older females |
| Nishiyama et al. (2008) | Mixed-Sex | Healthy males (n=15, 26±+/-1) and females (n=23, 23±1+/-1) | IRI | FMD; macrovascular | ↔ FMD in upper extremities  ↓ lower extremity FMD in females |
| Luca et al. (2016) | Female-Only | Healthy females aged 22.9±+/-1 | IRI | FMD; macrovascular | ↓ FMD in early follicular phase |
| Van Guilder et al. (2020) | Female-Only | Healthy females aged 21±1 | IRI | FBF; microvascular | ↓ FBF during early follicular phase with preconditioning  ↓ FBF during early ovulatory phase with preconditioning |
| Lalande et al (2021) | Mixed-Sex | Premenopausal females tested during early follicular phase of their menstrual cycle (n=12) and males (n=22) with similar estradiol levels | IRI | FMD; macrovascular | ↓ FMD in both females and males |
| Delgado Spicuzza et al. (2023) | Female-Only | Premenopausal (n=11)  Early (4±1.6 years) postmenopausal (n=11)  Late (15±5.5 years) postmenopausal (n=11) | IRI | FMD; macrovascular | ↓ FMD in early-postmenopausal |
| Durschlag et al. (2024) | Female-Only | Premenopausal females tested during early follicular and late follicular (n=11, age 22±1) early follicular and mid-luteal (n= 13, age 24±3) with no pregnancy history Postmenopausal females with no pregnancy history (n=13, age 57±6) | IRI | FBF; microvascular | ↓ FBF in early follicular phase  ↓ FBF in postmenopausal group |
| **Perturbation: Acute Hyperglycemia** | | | | | |
| Williams et al. (2020). | Female-Only | Females (n=17) without hormonal contraceptive use (age 20±1) | Oral glucose tolerance challenge | PWV; macrovascular | ↔ PWV across any menstrual phase |
| Lee et al (2020) | Female-Only | Females (n=24) who were healthy (n=7), had impaired fasting glucose (n=9) or diabetes (n=8) | Oral glucose tolerance challenge | PWV; macrovascular | ↑PWV in healthy females; no changes in females with impaired fasting glucose or diabetes |
| Ceriello et al (2002) | Mixed-Sex | Middle-aged females and males with diabetes (45% female) and controls (40% female); menopause status not reported | Oral glucose tolerance challenge | FMD; macrovascular | ↓ FMD with OGTT and OGTT + high fat meal in controls and those with diabetes |
| Weiss et al (2008) | Mixed-Sex | Middle-aged females and males (62% female); menopause status not reported | Oral glucose tolerance challenge | FMD; macrovascular | ↓ FMD with glucose test; improvement with exercise conducted 17h prior to glucose challenge |
| Har et al. (2014) | Mixed-Sex | Males and females (48% female) with type 1 diabetes (age ≥18 years) | Clamped hyperglycemia | FMD; macrovascular | ↓ FMD, magnitude greater females > males  ↑ angiotensin II in males |
| Williams et al. (2019). | Female-Only | Females (n=17) without cardiovascular or metabolic disease or hormonal contraceptive use (age 20-23) | Oral glucose tolerance challenge | FMD; macrovascular | ↓ FMD 90 minutes after perturbation, unaffected by menstrual cycle phase |
| Kralj et al (2025) | Mixed-Sex | Females (n=16) and males (n=16), mean age ~26 years | Oral glucose tolerance challenge | Microvascular function responses to ACh and SNP | ↓ Microvascular smooth muscle, but not endothelial, function response |
| **Perturbation: Acute Hyperlipidemia** | | | | | |
| Pincu et al. (2022) | Mixed-Sex | Control (no type 1 diabetes) adolescents (n=21, 57% female)  Type 1 diabetic (n=14, 42% female) adolescents aged 15-20 | High fat (59g) meal | PWV; macrovascular | ↔ PWV between diabetic groups  ↔ PWV after high fat meal |
| Vogel et al. (1997) | Mixed-Sex | Healthy adults (n=10, 50% females) aged 39±10 | High fat (50g) meal  Low fat (0g) meal | FMD; macrovascular | ↓FMD after high fat meal |
| Raitakari et al. (2000). | Mixed-Sex | Healthy adults (n=12, 41% female) aged 18-45 | High fat (61g) meal | Baseline blood flow; microvascular  Reactive hyperemia (RH); microvascular  FMD; macrovascular  Resting artery diameter; macrovascular | ↔ FMD after high fat meal  ↑ all other outcomes after high fat meal |
| Bae et al. (2000) | Mixed-Sex | Healthy males and postmenopausal females (n=20, 50% females) aged 56±12 | High fat (53.4g) meal | FMD; macrovascular | ↓FMD after high fat meal |
| Schillaci et al. (2001) | Female-Only | Healthy females (n=10) aged 23±2 | High fat (65g) meal | FMD; macrovascular | ↔ FMD after high fat meal |
| Ceriello et al (2002) | Mixed-Sex | Middle-aged females and males with diabetes (45% female) and controls (40% female); menopause status not reported | High fat meal | FMD; macrovascular | ↓ FMD with high fat meal and OGTT + high fat meal in controls and those with diabetes |
| Barringer et al (2011) | Mixed-Sex | Healthy adults (n=23; 78% female) | High fat (56g) meal | RH-PAT; microvascular | ↓RH-PAT after high fat meal |
| Harris et al. (2012) | Mixed-Sex | Healthy males and premenopausal females (n=25, 60% females) aged 19-23 | High fat (48g) meal | FMD; macrovascular | ↔ FMD after high fat meal in females  ↓FMD in males after high fat meal |
| Muniyappa et al. (2012) | Female-Only | Healthy (n= 38) females aged 37±1 | High fat (40%) meal | FMD; macrovascular | ↑ FMD after high fat meal  ↔ Postprandial FMD between African-American and White females |
| Marinos et al. (2015) | Female-Only | Obese females (n=17) aged 42±11 | High fat (83%) meal | FMD; macrovascular | ↓FMD after high fat meal |
| Swift et al (2015) | Female-Only | Postmenopausal females, n=8 African American, n=8 Caucasian | High fat meal (57% fat) | FMD; macrovascular | ↔ FMD after high fat meal in either group |
| **Perturbation: Cold Pressor Test** | | | | | |
| Gentilin et al. (2025) | Mixed-Sex | Healthy adults (n=28) (50% females) aged 18-25 | CPT | PWV; macrovascular  TPR | ↔ sex difference in PWV  ↑TPR in males |
| Prodel et al. (2018) | Mixed-Sex | Healthy adults (n=29) (44% females) aged 21-32 | CPT | AIx; macrovascular  BP  Heart rate | No changes to heart rate  Similar BP increases |
| McLean et al. (1991) | Mixed-Sex | Healthy adults (n=20, 50% female) aged 20-26 | CPT (2 methods, hand and face) | SBP  Norepinephrine levels | ↑ increase in SBP from rest in males with both CPT methods  No sex difference in norepinephrine |
| Fatima et al. (2020) | Mixed-Sex | Healthy adults (n=104, 57% female) aged 18-30 | CPT | SBP  DBP | ↑ increase in SBP from rest in males |
| Vandavasi and Sukumar et al. (2016) | Mixed-Sex | Healthy adults (n=60, 50% females) aged 19-25 | CPT | SBP  HR | ↑ SBP, HR, in females > males |
| Kilgour, R.D and Carvalho, J. (1993) | Mixed-Sex | Normotensive adults (n=18, 50% females) aged 20-32 | CPT | TPR  SBP  DBP | Greater and prolonged ↑ SBP,  ↑ TPR in males  ↑ HR in females |
| Gentilin et al. (2022) | Mixed-Sex | Healthy adults (n=25, 52% females in early follicular phase) aged 18-25 | CPT | FMD; macrovascular  TPR | ↑ FMD in females prior to normalization for baseline diameter and shear rate, similar after  ↑ TPR in males |
| Moro et al. (2011) | Mixed-Sex | Healthy adults (n=24, 50% females) aged 18-26 | CPT | Myocardial blood flow (MBF); microvascular | Greater ↑ MBF in females |
| Jacob et al. (2022) | Female-Only | Premenopausal, healthy females (n= 21), aged 18-45, during low estradiol (early follicular or placebo pill) and high estradiol (late follicular or combined oral contraceptive pill (OCP)) | CPT | FBF; microvascular | ↑ FBF in greatest in active OCP, placebo OCP, and late follicular  ↓FVC in early follicular |
| Jasrotia et al. (2017) | Female-Only | Healthy females (n=75), aged 19-27 during menstrual, follicular, and luteal phases | CPT | Systolic blood pressure reactivity (SBPR)  Diastolic blood pressure reactivity (DBPR) | ↑SBPR, DBPR in luteal phase |
| **Perturbation: Mental Stress Test** | | | | | |
| Logan et al. (2020) | Female-Only | Healthy premenopausal females (n=85) aged 18-55 | Trier Social Stress Test (TSST) | PWV; macrovascular  AIx; macrovascular  SBP, DBP, HR  Pre-ejection period (PEP) | ↑ PWV, AIx, SBP, DBP, HR after TSST  No significant PEP changes,  and not associated with PWV, AIx |
| Gentilin et al. (2023) | Mixed-Sex | Healthy adults (n=26, 50% female) aged 21-26 | Math test | TPR  PWV; macrovascular  HR  Pulse pressure (PP) | ↑PWV, TPR, HR  No effect of sex on PWV  ↑TPR females > males  ↑PP females < males |
| Chen et al. (2012) | Mixed-Sex | Healthy adolescents (n=162, 71% female) aged 15-19 and healthy children (n= 241, 48% female) aged 7-12 | Math test | PWV; macrovascular | ↑PWV  ↑PWV females < males  PWV not affected by age |
| Lipman et al (2002) | Mixed-Sex | Healthy adults (n=24, 67% female) aged 51-86 | Speech or math task | Stiffness (via vessel diameter measured from ultrasound images)  HR  Baroreflex sensitivity (BS) | No stiffness, HR, BS changes with aging  CS inversely related to HR  HR correlated with stiffness |
| Sarabi and Lind (2001) | Mixed-Sex | Healthy adults (n=10, 60% female) aged 20-25 undergoing infusions of methacholine and sodium nitroprusside | Math test | Index of endothelial function (IEF), based on forearm vascular resistance | ↓IEF |
| Harris et al. (2000) | Mixed-Sex | Healthy adults (n=21, 52% female) with average age of 23.5 years | Math test | FMD; macrovascular | ↑FMD  FMD not impacted by sex |
| Vaccarino et al. (2018) | Mixed-Sex | Healthy adults (n=112, 52% female, 35% premenopausal) aged ≤61 years | Speech task | FMD; macrovascular  RH; microvascular | ↓FMD  FMD not impacted by sex  ↑ RHI in females |
| Lind et al. (2002) | Mixed-Sex | Healthy adults (n=18, 44% female) aged 20-25 | Math test | FMD; macrovascular  Brachial artery diameter and blood flow (BABF); microvascular | ↔ FMD  ↑BABF |
| Martin et al. (2008) | Mixed-Sex | Healthy adults (n=87, 61% female, menopausal status unknown) aged 35-39 | Spiral omnibus, math test, word-color conflict | Reactive hyperemia peripheral arterial tonometry (RH-PAT); microvascular  Double product: systolic blood pressure x heart rate (DP) | ↑DP females < males  ↑RH-PAT in males, ↓ in females  ↓RH-PAT females with worse vasoreactivity < females with better vasoreactivity |
| Sullivan et al. (2017) | Mixed-Sex | Healthy adults (n=678, 27% female) aged 55-69 with follow-up for development of mental stress induced myocardial ischemia (MSIMI) | Speech task | Reactive hyperemia peripheral arterial tonometry (RH-PAT); microvascular  Double product: systolic blood pressure x heart rate (DP) | DP not impacted by sex  ↑DP in males after MSIMI  RH-PAT not impacted by sex  ↑RH-PAT in females after MSIMI |
| Lima et al (2019) | Mixed-Sex | Patients with coronary artery disease (n=569, 26% female) aged 63 ± 9 | Speech task | FMD; macrovascular | ↓FMD |
| D’Urzo et al. (2019) | Female-Only | Healthy females (n=43) aged 33±14 with a diagnosed depressive disorder | Trier social stress test (TSST) | FMD; macrovascular | ↔ FMD |

**Supplementary Table 1:** Characteristics of studies examining mixed-sex or female-specific populations only assessing impact of perturbations on measurements of endothelial function and arterial stiffness. Perturbations included ischemia-reperfusion injury (IRI). Outcomes included forearm blood flow (FBF), forearm vascular conductance (FVC), flow-mediated dilation (FMD), augmentation index (AIx), pulse wave velocity (PWV), systolic blood pressure (SBP), diastolic blood pressure (DBP), heart rate (HR), index of endothelial function (IEF), brachial artery diameter and blood flow (BABF) , and total peripheral resistance (TPR).
